# *GLIS3* rs7034200 and *ADRB3* rs4994 genetic variants associated with an increased risk of gestational diabetes mellitus in Chinese women: a case-control study

**DOI:** 10.1186/s12884-025-08436-9

**Published:** 2025-11-21

**Authors:** Liping Shui, Qingqing Liu, Xinghui Liu, Huai Bai, Yujie Wu, Yuting Sheng, Guolin He, Ping Fan

**Affiliations:** 1 Department of Obstetrics and Gynecology, West China Second University Hospital, Sichuan University, Chengdu, Sichuan China; 2 Laboratory of Genetic Disease and Perinatal Medicine, Laboratory of the Key Perinatal Disease, Key Laboratory of Birth Defects and Related Disease of Women and Children, West China Second University Hospital, Ministry of Education, Sichuan University, Chengdu, Sichuan China

**Keywords:** GLIS3, ADRB3, Genetic polymorphism, Gestational diabetes mellitus, Glucolipid metabolism, Oxidative stress

## Abstract

**Background:**

Gestational diabetes mellitus (GDM) has a complicated pathophysiology originating from interactions between susceptibility genes and adverse environmental factors. The transcription factor GLI-similar 3 (*GLIS3*) rs7034200C/A and adrenergic receptor beta 3 (*ADRB3*) rs4994T/C (Trp64Arg) polymorphisms are closely related to glucolipid metabolism, insulin resistance, obesity, and increased risk of type 2 diabetes. However, the association of these two polymorphisms with GDM in Chinese remains unknown. Therefore, this study explored the relationship between these two genetic variants and the risk of GDM, and assessed the effects of genotypes on glucolipid metabolism, oxidative stress, and clinical indicators.

**Methods:**

This case-control study included 701 women with GDM and 1034 controls. The rs7034200C/A and rs4994T/C variants were genotyped using polymerase chain reaction-restriction fragment length polymorphism method. Clinical and biochemical parameters were analyzed.

**Results:**

The *GLIS3* rs7034200C/A polymorphism was associated with an elevated risk of GDM according to the genotype, allele, dominant, and recessive genetic models (*P* < 0.05). After correcting for maternal age, pre-pregnancy BMI, and gestational age at sampling, the dominant and recessive models retained statistical significance in the binary logistic regression models (odds ratio [OR] = 1.268 and 1.334, 95% confidence interval [CI]: 1.017–1.580 and 1.020–1.745, respectively; *P* < 0.05) ; the AA genotype showed a higher risk for GDM, with the CC genotype as the reference, in a multinomial logistic regression model (OR = 1.519, 95% CI: 1.120–2.061; *P* = 0.007). No significant discrepancies were observed in the *ADRB3* rs4994T/C polymorphism based on different genetic models (*P* > 0.05). However, the AA/TT combined genotype of the *GLIS3* rs7034200C/A and *ADRB3* rs4994T/C polymorphisms was associated with an elevated risk of GDM, with the CC/TT combined genotype as a reference (OR = 1.708, 95% CI: 1.191–2.450; *P* = 0.004). Furthermore, the *GLIS3* rs7034200C/A variant significantly affected oxidative stress, insulin resistance, and glucolipid metabolism.

**Conclusions:**

The A allele and AA genotype of the *GLIS3* rs7034200C/A polymorphism, along with the AA/TT combined genotype of the *GLIS3* rs7034200C/A and *ADRB3* rs4994T/C variants, are genetic risk factors for GDM in Chinese women.

**Supplementary Information:**

The online version contains supplementary material available at 10.1186/s12884-025-08436-9.

## Background

Gestational diabetes mellitus (GDM) is a glucose metabolism abnormality of varying degrees that first occurs or is diagnosed in the middle or late stages of pregnancy [1, 2]. With the increasing ratio of women with advanced maternal age and overweight/obesity, along with the influence of unhealthy lifestyles and adverse environmental factors, the current global prevalence of GDM ranges from 6.6% to 45.3% across different populations. It is rising annually [3], with an approximate incidence of 14.8% among Chinese women [4]. GDM adversely affects both mothers and offspring, with near-term complications, such as fetal malformations, macrosomia, neonatal hypoglycemia, pre-eclampsia, and higher cesarean section rates, and long-term outcomes, including increased susceptibility to metabolic syndrome, type 2 diabetes (T2D), and cardiovascular events [1, 2, 5]. The etiopathogenesis of GDM remains unclear, but growing evidence suggests that increased pre-pregnancy body mass index (BMI), genetic variants, redox disequilibrium, chronic inflammation, dyslipidemia, the use of assisted reproductive technology, and/or pancreatic β-cell damage are associated with its development [2, 6–10].

The transcription factor GLI-similar 3 (GLIS3) is encoded by a gene (*GLIS3*) located on chromosome 9 (9p24.2) and is highly expressed in the thyroid, pancreas, and kidney [11, 12]. GLIS3 can regulate multiple biological processes, including the formation of pancreatic β-cells, transcription of the insulin gene, thyroid development and hormone synthesis, maintenance of normal renal structure and function, and spermatogenesis [11, 12]. Functional defects caused by *GLIS3* mutations result in neonatal diabetes syndrome, infertility, and polycystic kidney disease [12]. Multiple single nucleotide polymorphisms (SNPs) in *GLIS3* have been associated with the genetic predisposition of several diseases, including type 1 diabetes, T2D, neurological disorders, and glaucoma, in diverse populations [11–13]. Among these SNPs, the *GLIS3* rs7034200C/A variant has been related to β-cell function, fasting glucose levels, and the risk of T2D [11–18].

The adrenergic receptor beta 3 (ADRB3), a G protein-coupled receptor mainly expressed in adipocytes, is activated by catecholamines (noradrenaline and adrenaline) and contributes to lipolysis, thermogenesis, and body fat distribution [19, 20]. Human *ADRB3* is located on chromosome 8 (8p11.23) [19]. The expression of *ADRB3* in adipose tissue is significantly lower in overweight and obese individuals [21, 22]. The *ADRB3* rs4994T/C (Trp64Arg) SNP, a missense variant, has a C allele that has been linked to lower resting metabolic rates, insulin resistance, and elevated risk of overweight/obesity and T2D [19, 21, 23].

Insulin resistance and pancreatic β-cell dysfunction play critical roles in the onset and progression of GDM. The *GLIS3* SNPs rs7041847 and rs10814916 are associated with an elevated risk of GDM [24], whereas the rs7034200 SNP is linked to a reduced risk of GDM in Caucasian populations [14]. However, it remains unclear whether the *ADRB3* rs4994 and *GLIS3* rs7034200 genetic polymorphisms are associated with GDM in Chinese population. Based on the findings that these two SNPs are closely related to glucolipid metabolism, insulin resistance, obesity, and increased risk of T2D [11, 16, 18, 19], we hypothesized that they may be involved in the risk of GDM development among Chinese. Therefore, we investigated the correlation between these two polymorphisms and the genetic predisposition of GDM, and analyzed the effects of these variants on glucolipid metabolism, oxidative stress, and clinical characteristics among pregnant Chinese women.

## Methods

### Study participants

This case-control study included 701 patients with GDM and 1,034 healthy pregnant women. The study participants were pregnant women who were registered at the obstetrics outpatient clinic of the West China Second Hospital from 2013 to 2021. Detailed recruitment process of cases and controls was depicted in Supplementary Fig. 1. All participants gave their written informed consent. This study was approved by the Ethics Committee of the West China Second Hospital of Sichuan University (Xinghui Liu, No. 2017−033 and Ping Fan, No. 2020-036) and was performed in accordance with the Declaration of Helsinki.

The enrolled pregnant women underwent a standard 75-g oral glucose tolerance test (OGTT) between 24 and 28 weeks of pregnancy. GDM was diagnosed based on the criteria of the International Association of Diabetes and Pregnancy Study Groups [25]. Healthy control pregnant women were concurrently recruited from the same hospital.

In this study, each pregnant woman was subjected to standardized and good pregnancy health care, and had not taken any other medications except vitamins and minerals for pregnant women (Elevit) and calcium (Caltrate) regularly during pregnancy. Based on ACOG guidelines for GDM [26], each woman diagnosed with GDM received nutrition and exercise counseling, carried on a routine monitoring of fasting and 2-h postprandial blood glucose, and when this failed to adequately control glucose levels, insulin should be used for maternal and fetal benefit.

The exclusion criteria of the participants were twin/multiple pregnancies; pre-pregnancy diabetes; intrahepatic cholestasis of pregnancy; chronic hypertension; pre-eclampsia; gestational hypertension; smoking; and other autoimmune, endocrine, hepatic, cardiac, and renal diseases. Additionally, participants who had undergone in vitro fertilization, or those with preterm labor, a history of GDM were excluded from the control group.

Clinical data, including gestational age, blood pressure, pre-pregnancy and delivery BMI (kg/m^2^), gestational age at sampling, neonatal weight and length, and OGTT result in the second trimester, were recorded. A neonate with a birth weight of ≥ 4,000 g was defined as macrosomia [27].

Fasting venous blood was obtained in the late trimester or before birthing. Blood cells were preserved at 4 °C for DNA extraction. Plasma and serum were packaged separately and stored at − 80 °C for subsequent testing and analysis.

### Analysis of genotypes

**Fig. 1:**
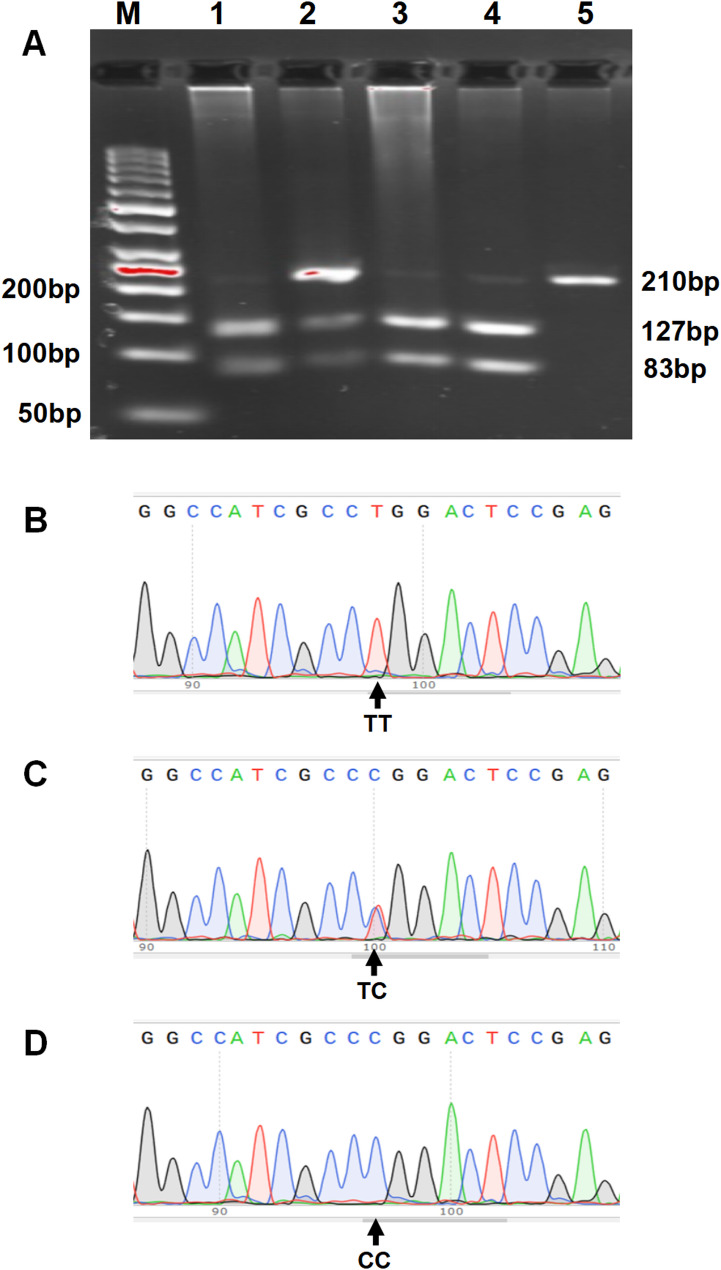
Polymerase chain reaction-restriction fragment length polymorphism analysis and sequencing validation of *ADRB3* rs4994T/C genetic polymorphism. **A** The 3 patterns represent the T- and C-containing genotypes, namely, the homozygous TT and CC forms and the heterozygous CT form. Lane M shows the 50 bp DNA ladder; lanes 1, 3, and 4 show the TT genotype; lane 2 shows CT genotype, and lane 5 shows CC genotype. **B**‒**C** The DNA sequencing validation of the amplified PCR products: (**B**) wild-type, TT homozygote; (**C**) TC heterozygote variant; (**D**) CC homozygote variant

**Fig. 2:**
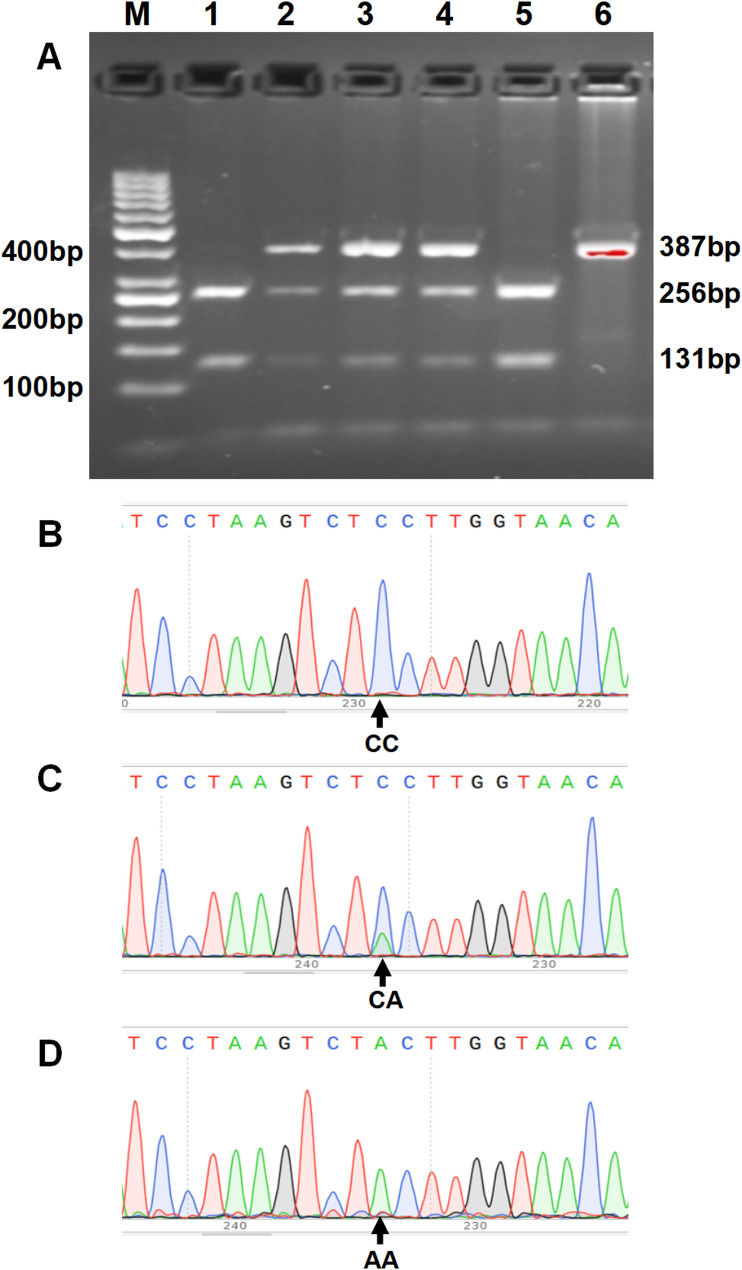
Polymerase chain reaction-restriction fragment length polymorphism analysis and sequencing validation of the *GLIS3* rs7034200C/A genetic variant. **A** The 3 patterns represent the C- and A-containing genotypes, namely, the homozygous CC and AA forms and the heterozygous CA form. Lane M shows the 50 bp DNA ladder; lanes 1 and 5 show the CC genotype; lane 2, 3, and 4 show CA genotype, and lane 6 shows AA genotype. **B**‒**C** The DNA sequencing validation of the amplified PCR products: (**B**) wild-type, CC homozygote; (**C**) CA heterozygote variant; (**D**) AA homozygote variant

Genomic DNA was purified from stored blood cells and the genotypes of *ADRB3* rs4994T/C and *GLIS3* rs7034200C/A SNPs were determined by polymerase chain reaction-restriction fragment length polymorphism analysis and DNA sequencing validation. The primers of the *ADRB3* (gene ID: 155) rs4994 SNP referred to the report by Widen et al. [28], with an upstream primer: 5’- CGCCCAATACCGCCAACAC-3’ and a downstream primer: 5’-CCACCAGGAGTCCCATCACC-3’. The primers of the *GLIS3* (gene ID: 169792) rs7034200 SNP were designed using Primer-BLAST (upstream primer: 5’-TGATTATAGAGCAAATGGGGTGTTC-3’, downstream primer: 5’-TAATTACGCCAACAGATTTCTC-3’). After PCR amplification, a 3.5 µL PCR products of the rs4994 SNP (210 bp) or 5 µL rs7034200 SNP (387 bp) was digested using 3.5 U of PflMI and 2 U of BsaJI (New England Biolabs, Inc.), respectively. Digestion produced 127- and 83-bp fragments confirming the rs4994T allele, an undigested 210-bp fragment confirming the rs4994C allele (Fig. 1A), 256- and 131-bp fragments confirming the rs7034200C allele, and an undigested 387-bp fragment confirming the rs7034200A allele (Fig. 2A). The digested products were visualized using 3.5 or 2.5% agarose gel electrophoresis with Genecolor fluorescent dye. In addition, a DNA sequencing validation of amplified PCR products was conducted for *ADRB3* rs4994T/C (Fig. 1B-C) and *GLIS3* rs7034200C/A (Fig. 2B-C) SNPs. More than 30% of the randomly selected specimens were verified by another researcher for quality evaluation, and the two results were consistent.

### Analysis of biochemical indicators

Plasma insulin levels were determined by chemiluminescence assays (Immulite 2000; Diagnostic Products Corporation, Los Angeles, CA, USA). Plasma glucose concentrations were tested by the glucose oxidase technique (Roche Diagnostics GmbH, Mannheim, Germany). Serum apolipoprotein (apo)B and apoA1 levels were measured using the polyethylene glycol (PEG) enhanced immunoturbidimetric assay (Siemens Healthcare Diagnostics Inc., Tarrytown, NY 10591 − 5097 USA). Total cholesterol (TC), low-density lipoprotein cholesterol (LDL-C), high-density lipoprotein cholesterol (HDL-C), and triglycerides (TG) levels were determined using enzymatic assay (Siemens Healthcare Diagnostics Inc., Tarrytown, NY 10591 − 5097 USA). The homeostatic model assessment of insulin resistance (HOMA-IR) was calculated as previously described [29] using the following formula:

HOMA-IR = fasting glucose (mmol/L) × fasting insulin (µU/ml)/22.5.

Serum malondialdehyde (MDA) concentrations were measured colorimetrically by the thibabituric acid reactive substance method with 1,3,3,3-tetraethoxypropane as a standard using micro-MDA kits (NanJing Jiancheng Bioengineering Institute, Nan Jing, China). Serum total oxidative status (TOS) and total antioxidant capacity (TAC) were measured using the semi-automatic microplate colorimetric method as previously described [30, 31]. The measurement of TOS was based on the oxidation of Fe^2+^ to Fe^3+^ in the presence of the oxidants contained in serum using hydrogen peroxide (H_2_O_2_) as a standard [30]. The determination of TAC is based on the reaction of 2,2-azino-bis-3-ethylbenzothiazoline-6-sulphonic acid radical cation (ABTS**˙**^**+**^) with antioxidants present in serum using Trolox (6-hydroxy-2,5,7,8-tetramethylchroman-2-carboxylic acid) as a calibration [31]. Oxidative stress index (OSI, arbitrary unit) expressed the ratio of TOS to TAC [31, 32].

For all assays, the intra-lot variation was < 5% and the inter-lot variation was < 10%.

### Statistical analyses

All data were analyzed using the Statistical Program for Social Sciences (SPSS) 21.0 (IBM SPSS Statistics, IBM Corporation). Hardy–Weinberg equilibrium, genotype distribution, and allele frequencies were analyzed in the GDM and control groups using chi-square tests. Odds ratios (ORs) and 95% confidence intervals (CIs) were used to estimate the relative risk of GDM associated with the genetic variants, using chi-square tests and logistic regression analysis. Independent sample t-tests, Mann-Whitney U tests, or analysis of variance were used to assess the differences in continuous variables between the two groups or subgroups. Adjustment for confounding factors was performed using Analysis of covariance. Statistical significance was set at *P* < 0.05.

Power values were calculated according to the disease (A) allele frequency of the *GLIS3* rs7034200C/A variant and sample size using an online Genetic Association Study Power Calculator (http://csg.sph.umich.edu/abecasis/GAS_power_Calculator/index).

## Results

### Clinical and biochemical characteristics of the participants

Significant discrepancies in pre-pregnancy BMI, gestational age at sampling, and maternal age between the control and GDM groups (Table 1) or the genotype subgroups (*GLIS3* rs7034200 SNP) in this study prompted us to correct these three confounding factors in our subsequent analyses.

**Table 1:** Clinical and biochemical characteristics of women with and without GDM

	Controls (*n* = 1034)	GDM (*n* = 701)	*P*	*P* ^a^
Clinical characteristics				
Age (year)	35.46 ± 3.75	35.56 ± 4.08	0.600	
Pre-pregnancy BMI (kg/m^2^)	21.20 ± 2.69	22.25 ± 2.96	< 0.001	
Delivery BMI (kg/m^2^)	26.69 ± 2.70	26.83 ± 3.19	0.350	
Gestational age at sampling (wk)	36.12 ± 3.53	37.02 ± 3.20	< 0.001	
Gestational age (wk)	39.27 ± 0.86	38.96 ± 1.06	< 0.001	< 0.001
Pregnancy weight gain (kg)	14.00 ± 4.30	11.51 ± 4.16	< 0.001	< 0.001
SBP (mmHg)	114.97 ± 10.11	115.81 ± 11.21	0.103	0.468
DBP (mmHg)	72.17 ± 7.07	72.72 ± 8.88	0.185	0.515
OGTT-fasting Glu (mmol/L)*	4.42 ± 0.29	4.88 ± 0.53	< 0.001	< 0.001
OGTT-1 h Glu (mmol/L)*	7.45 ± 1.28	9.89 ± 1.38	< 0.001	< 0.001
OGTT-2 h Glu (mmol/L)*	6.53 ± 1.02	8.68 ± 1.34	< 0.001	< 0.001
Neonatal birth height (cm)	49.88 ± 1.88	49.60 ± 1.85	0.003	0.001
Neonatal birth weight (g)	3386.38 ± 368.32	3334.36 ± 443.36	0.011	< 0.001
Macrosomia % (n)	4.3 (44)	5.1 (36)	0.391	
Insulin treatment (n)	0	74		
Metabolic parameters**				
Fasting Glu (mmol/L)	4.35 ± 0.43	4.60 ± 0.73	< 0.001	< 0.001
Fasting Ins (pmol/L)	72.04 ± 35.13	104.32 ± 129.18	< 0.001	< 0.001
HOMA-IR	2.03 ± 1.09	3.41 ± 5.74	< 0.001	< 0.001
TG (mmol/L)	3.62 ± 1.40	3.90 ± 1.67	0.001	0.022
TC (mmol/L)	6.06 ± 1.09	5.95 ± 1.09	0.047	0.135
HDL-C (mmol/L)	1.99 ± 0.42	1.97 ± 0.43	0.297	0.640
LDL-C (mmol/L)	3.18 ± 1.00	2.97 ± 0.97	< 0.001	< 0.001
TG/HDL-C	1.91 ± 0.88	2.10 ± 1.10	< 0.001	0.014
ApoA1 (g/L)	2.37 ± 0.41	2.30 ± 0.42	0.001	0.031
ApoB (g/L)	1.15 ± 0.26	1.15 ± 0.26	0.964	0.786
Oxidative stress parameters***				
TOS (µmol H_2_O_2_ Equiv./L)	21.18 ± 7.03	25.89 ± 10.56	< 0.001	< 0.001
TAC (mmol Trolox Equiv./L)	1.10 ± 0.19	1.12 ± 0.21	0.262	0.556
OSI	19.57 ± 7.29	23.34 ± 10.11	< 0.001	< 0.001
MDA (µmol/L)	5.38 ± 1.21	5.88 ± 1.42	< 0.001	< 0.001

Values are presented as mean ± SD unless otherwise noted

*GDM* Gestational diabetes mellitus, *BMI* Body mass index, *SBP* Systolic blood pressure, *DBP* Diastolic blood pressure, *OGTT* Oral glucose tolerance test, *Glu* Glucose, *Ins* Insulin, *HOMA-IR* Homeostatic model assessment of insulin resistance, *TG* Triglyceride, *TC* Total cholesterol, *HDL-C* High-density lipoprotein cholesterol, *LDL-C* Low-density lipoprotein cholesterol, *ApoA1* Apolipoprotein A1, *apoB* Apolipoprotein B, *TOS* Total oxidant status, *TAC* Total antioxidant capacity, *OSI* Oxidative stress index, *MDA* Malondialdehyde

^a^All parameter comparisons were corrected for differences in age, pre-pregnancy BMI, and gestational age at sampling between the two groups, except for age, BMIs, and gestational age at sampling

^*^Plasma glucose levels during OGTT between 24 and 28 weeks of gestation

^**^Controls (*n* = 972), GDM (*n* = 643)

^***^Controls (*n* = 760), GDM (*n* = 533)

Table 1 shows that patients with GDM had significantly higher fasting, 1-h, and 2-hour glucose concentrations in the OGTT at 24–28 weeks of pregnancy, along with higher fasting glucose and insulin, TG, TG/HDL-C ratio, HOMA-IR, TOS, MDA, and OSI, but lower gestational age, pregnancy weight gain, neonatal birth height and weight, and apoA1 and LDL-C levels compared to controls (*P* < 0.05). Among the 701 patients, 74 required insulin therapy, and the remainder were treated with exercise or dietary modifications. No significant discrepancy in the proportion of macrosomia was observed between the control and GDM groups (*P* > 0.05).

### Genotypic and allelic frequencies of *GLIS3* rs7034200C/A and *ADRB3* rs4994T/C SNPs

Different genetic models for the *GLIS3* rs7034200 and *ADRB3* rs4994 SNPs are summarized in Table 2. The genotypic distributions of both SNPs conformed to Hardy-Weinberg equilibrium in the GDM and control groups (all *P* > 0.05).

**Table 2:** Association of *GLIS3* rs7034200C/A and *ADRB3* rs4994T/C polymorphisms with the risk of GDM using different genetic models

	Controls (*n* = 1034)	GDM (*n* = 701)	χ2	*P*	OR	95% CI
***GLIS3*** rs7034200						
Genotype						
CC	351 (33.9%)	201 (28.7%)	referent			
CA	526 (50.9%)	363 (51.8%)	2.792	0.095	1.205	0.968–1.500
AA	157 (15.2%)	137 (19.5%)	8.296	0.004	1.524	1.143–2.031
*P*_HWE_	0.215	0.502				
Recessive						
CA + CC	877 (84.8%)	564 (80.5%)				
AA	157 (15.2%)	137 (19.5%)	5.642	0.018	1.357	1.054–1.746
Dominant						
CC	351 (33.9%)	201 (28.7%)				
CA + AA	683 (66.1%)	500 (71.3%)	5.354	0.021	1.278	1.038–1.574
Allele						
C	1228 (59.4%)	765 (54.6%)				
A	840 (40.6%)	637 (45.4%)	7.928	0.005	1.217	1.062–1.396

***ADRB3*** rs4994						
Genotype						
TT	753 (72.8%)	505 (72.0%)	referent			
TC	260 (25.2%)	185 (26.4%)	0.279	0.597	1.061	0.852–1.322
CC	21 (2.0%)	11 (1.6%)	0.433	0.511	0.781	0.373–1.637
*P*_HWE_	0.966	0.438				
Recessive						
TC + TT	1013 (98.0%)	690 (98.4%)				
CC	21 (2.0%)	11 (1.6%)	0.4922	0.483	0.769	0.368–1.605
Dominant						
TT	753 (72.8%)	505 (72.0%)				
TC + CC	281 (27.2%)	196 (28.0%)	0.129	0.720	1.040	0.839–1.289
Allele						
T	1766 (85.4%)	1195 (85.3%)				
C	302 (14.6%)	207 (14.7%)	0.017	0.895	1.013	0.836–1.227

Genotype and allele data are presented as numbers (%)

*OR* Odds ratio, *CI* Confidence interval

*P*_HWE_, *P* value of Hardy-Weinberg equilibrium test

The frequencies of the A allele and AA genotype of the *GLIS3* rs7034200C/A variant were significantly higher in the GDM group (45.4% and 19.5%, respectively) than in the control group (40.6% and 15.2%, respectively), with an OR of 1.278 (95% CI: 1.038–1.574; *P* = 0.021) for the dominant model, 1.357 (95% CI: 1.054–1.746; *P* = 0.018) for the recessive model, 1.217 (95% CI: 1.062–1.396; *P* = 0.005) for the allele model, and 1.524 (95% CI: 1.143–2.031; *P* = 0.004) for the CC vs. AA genotypes. After adjusting for three confounding factors, the dominant and recessive models retained statistical significance in the binary regression models (OR = 1.268, 95% CI: 1.017–1.580; *P* = 0.035 and OR = 1.334, 95% CI: 1.020–1.745; *P* = 0.035, respectively); the AA genotype also showed a higher risk for GDM in a multinomial logistic regression model, with the CC genotype as the reference (OR = 1.519, 95% CI: 1.120–2.061; *P* = 0.007). Further adjusting for gestational weight gain along with glycolipid metabolic parameters (HOMA-IR, LDL-C, and TG/HDL-C ratio), the dominant, recessive and genotype models no longer remained statistical significance (*P* > 0.05; Supplementary Table 1). The genetic association power value was 0.944 (prevalence = 0.148, significance level = 0.05) for the adjusted recessive model of the *GLIS3* rs7034200 SNP.

No significant discrepancies were observed between the case and control groups based on the allele, genotype, dominant, or recessive models for *ADRB3* rs4994T/C polymorphism (*P* > 0.05, Table 2).

The results of the combined genotype analysis of the *GLIS3* rs7034200C/A and *ADRB3* rs4994T/C SNPs are shown in Table 3. The small number of CC genotypes in the *ADRB3* rs4994 SNP among cases and controls necessitated their combination with the heterozygous CT genotype subgroups. The combined AA/TT genotypic frequency was significantly higher in the case group (14.7%) than in the control group (10.3%). Multinomial logistic regression analysis indicated that it was associated with an elevated risk of GDM, with the combined CC/TT genotype as the reference and maternal age, gestational age at sampling, and pre-pregnancy BMI as covariates (OR = 1.708, 95% CI: 1.191–2.450; *P* = 0.004).

**Table 3:** Frequencies of combined genotypes of *GLIS3* rs7034200C/A and *ADRB3* rs4994T/C in women with and without GDM

	Controls (*n* = 1034)	GDM (*n* = 701)	OR	95% CI	*P* ^a^
*GLIS3* rs7034200C/A and *ADRB3* rs4994T/C*					
CC/TT	259 (25%)	146 (20.8%)	1.000	–	–
CA/TT	387 (37.4%)	256 (36.5%)	1.174	0.894–1.541	0.248
AA/TT	106 (10.3%)	103 (14.7%)	1.708	1.191–2.450	0.004
CC/CC + TC	92 (8.9%)	55 (7.8%)	1.028	0.682–1.548	0.896
CA/CC + TC	139 (13.4%)	107 (15.3%)	1.373	0.977–1.930	0.068
AA/CC + TC	51 (4.9%)	34 (4.9%)	1.169	0.705–1.939	0.545

Data on the combined genotypes are presented as the number (%) of women with and without GDM

^*^Chi-squared test: χ^2^ = 11.652, *P* = 0.040. Odds ratios (ORs) and 95% confidence intervals (CIs) were calculated using a multinomial logistic regression model, including age, pre-pregnancy BMI, and gestational age at sampling as covariates, and the CC/TT wild-type combined genotypes as the reference category

### Effects of *GLIS3* rs7034200C/A and *ADRB3* rs4994T/C variants on clinical and biochemical indicators

Table 4 shows that patients carrying the CA genotype of the *GLIS3* rs7034200C/A SNP had lower TOS levels (*P* = 0.008), OSI (*P* = 0.048), and BMI at delivery (*P* = 0.058) than those carrying the CC genotype and lower TOS levels than those carrying the AA genotype (*P* = 0.047). Controls carrying the AA genotype exhibited higher OGTT-fasting glucose concentrations (*P* < 0.01) and slightly increased OGTT-1 h glucose levels (*P* < 0.07) than those carrying the CC or CA genotype; higher OGTT-2 h glucose (*P* = 0.008), fasting insulin (*P* = 0.033), and HOMA-IR (*P* = 0.039) than those carrying the CC genotype; and higher TAC (*P* = 0.007) but lower BMI at delivery (*P* = 0.058) than those carrying the CA genotype. Controls with the CA genotype exhibited lower HDL-C levels (*P* = 0.040) but higher age and TG/HDL-C ratio (*P* < 0.007) than those with the CC genotype.

**Table 4:** Clinical and biochemical parameters according to *GLIS3* rs7034200C/A different genotypes in women with and without GDM

	Controls			GDM		
	CC (*n* = 351)	CA (*n* = 526)	AA (*n* = 157)	CC (*n* = 201)	CA (*n* = 363)	AA (*n* = 137)
Clinical characteristics						
Age (years)	35.13 ± 3.68	35.67 ± 3.81^a^	35.50 ± 3.72	35.43 ± 4.19	35.68 ± 3.99	35.45 ± 4.19
Pre-pregnancy BMI (kg/m^2^)	21.23 ± 2.43	21.22 ± 2.88	21.08 ± 2.56	22.38 ± 2.99	22.16 ± 2.98	22.32 ± 2.85
Delivery BMI (kg/m^2^)	26.65 ± 2.50	26.82 ± 2.87	26.35 ± 2.54	27.19 ± 3.79	26.66 ± 2.93	26.76 ± 2.88
Gestational age at sampling (wk)	36.27 ± 3.51	36.11 ± 3.53	35.85 ± 3.57	37.14 ± 3.21	36.90 ± 3.23	37.17 ± 3.12
Gestational age (wk)	39.25 ± 0.88	39.28 ± 0.85	39.29 ± 0.77	39.08 ± 0.86	38.92 ± 1.07	38.88 ± 1.26
Pregnancy weight gain (kg)	13.75 ± 4.01	14.28 ± 4.53	13.62 ± 4.04	11.84 ± 4.46	11.44 ± 3.97	11.17 ± 4.21
SBP (mmHg)	114.21 ± 10.50	115.28 ± 9.94	115.61 ± 9.77	115.23 ± 13.01 0.01	116.22 ± 10.51	115.60 ± 10.14
DBP (mmHg)	72.04 ± 7.92	72.10 ± 7.65	72.67 ± 7.42	72.54 ± 10.17	73.06 ± 8.33	72.08 ± 8.30
OGTT-fasting Glu (mmol/L)*	4.41 ± 0.29	4.41 ± 0.29	4.49 ± 0.29^a, b^	4.87 ± 0.52	4.91 ± 0.55	4.86 ± 0.51
OGTT-1 h Glu (mmol/L)*	7.40 ± 1.34	7.43 ± 1.26	7.63 ± 1.23	9.83 ± 1.32	9.94 ± 1.41	9.81 ± 1.38
OGTT-2 h Glu (mmol/L)*	6.42 ± 1.04	6.56 ± 1.00	6.69 ± 1.04^a^	8.55 ± 1.44	8.72 ± 1.35	8.75 ± 1.12
Neonatal birth height (cm)	49.83 ± 1.95	49.88 ± 1.89	49.99 ± 1.64	49.67 ± 1.92	49.60 ± 1.76	49.51 ± 1.96
Neonatal birth weight (g)	3379.23 ± 374.95	3389.50 ± 370.30	3391.91 ± 348.44	3311.30 ± 410.72	3345.50 ± 441.75	3338.76 ± 492.83
Metabolic parameters**						
Fasting Glu (mmol/L)	4.33 ± 0.43	4.38 ± 0.43	4.34 ± 0.42	4.59 ± 0.71	4.60 ± 0.74	4.64 ± 0.77
Fasting Ins (pmol/L)	69.94 ± 34.48	71.98 ± 35.32	75.56 ± 35.92^a^	99.66 ± 125.88	103.64 ± 132.97	113.47 ± 124.30
HOMA-IR	1.96 ± 1.04	2.05 ± 1.12	2.15 ± 1.09^a^	3.25 ± 6.20	3.39 ± 5.65	3.72 ± 5.24
TG (mmol/L)	3.52 ± 1.21	3.67 ± 1.52	3.69 ± 1.39	3.79 ± 1.74	3.93 ± 1.66	4.00 ± 1.60
TC (mmol/L)	6.12 ± 1.12	6.01 ± 1.10	6.09 ± 1.12	5.94 ± 1.01	5.95 ± 1.11	5.96 ± 1.17
HDL-C (mmol/L)	2.02 ± 0.44	1.96 ± 0.41^a^	2.00 ± 0.36	1.97 ± 0.43	1.96 ± 0.44	1.99 ± 0.44
LDL-C (mmol/L)	3.22 ± 0.98	3.16 ± 1.05	3.15 ± 0.87	2.96 ± 0.89	2.98 ± 1.04	2.93 ± 0.90
TG/HDL-C	1.83 ± 0.80	1.96 ± 0.94^a^	1.91 ± 0.84	2.06 ± 1.32	2.12 ± 1.03	2.10 ± 0.91
ApoA1 (g/L)	2.37 ± 0.39	2.36 ± 0.42	2.41 ± 0.44	2.30 ± 0.40	2.30 ± 0.45	2.28 ± 0.37
ApoB (g/L)	1.16 ± 0.27	1.15 ± 0.26	1.15 ± 0.25	1.15 ± 0.26	1.16 ± 0.26	1.14 ± 0.27
Oxidative stress parameters***						
TOS (µmol H2O2 Equiv./L)	20.72 ± 6.77	21.38 ± 7.12	21.70 ± 7.39	27.13 ± 11.50	24.59 ± 9.90^c^	27.38 ± 10.39^d^
TAC (mmol Trolox Equiv./L)	1.11 ± 0.19	1.09 ± 0.19	1.14 ± 0.21^b^	1.13 ± 0.21	1.11 ± 0.21	1.12 ± 0.20
OSI	19.17 ± 6.81	19.93 ± 7.49	19.44 ± 7.75	24.24 ± 11.35	22.36 ± 9.42^c^	24.54 ± 9.66
MDA (µmol/L)	5.37 ± 1.13	5.39 ± 1.25	5.38 ± 1.22	5.80 ± 1.18	5.86 ± 1.50	6.07 ± 1.54

Values are presented as mean ± SD

*BMI* Body mass index, *DBP* Diastolic blood pressure, *SBP* Systolic blood pressure, *OGTT* Oral glucose tolerance test, *Glu* Glucose, *Ins* Insulin, *HOMA-IR* Homeostatic model assessment of insulin resistance, *TG* Triglyceride, *TC* Total cholesterol, *HDL-C* High-density lipoprotein cholesterol, *LDL-C* Low-density lipoprotein cholesterol, *apoA1* Apolipoprotein A1, *apoB* Apolipoprotein B, *TOS* Total oxidant status, *TAC* Total antioxidant capacity, *OSI* Oxidative stress index, *MDA* Malondialdehyde

All parameter comparisons were corrected for differences in age, pre-pregnancy BMI, and gestation at sampling between the two subgroups, except for age, BMIs, and gestational age at sampling

^a^*P* < 0.05: compared with the CC genotype subgroup in the control group

^b^*P* < 0.05: compared with the CA genotype subgroup in the control group

^c^*P* < 0.05: compared with the CC genotype subgroup in the GDM group

^d^*P* < 0.05: compared with the CA genotype subgroup in the GDM group

^*^Plasma glucose levels during OGTT between 24 and 28 weeks of gestation

^**^Controls (CC = 324, CA = 498, AA = 150); GDM (CC = 188, CA = 332, AA = 123)

^***^Controls (CC = 256, CA = 387, AA = 117); GDM (CC = 158, CA = 272, AA = 103)

However, no significant discrepancies in clinical, metabolic, or oxidative stress indicators were observed based on the different genotypes of the *ADRB3 *rs4994T/C SNP in the control and GDM groups (*P* > 0.05; Supplementary Table 2).

## Discussion

This study is the first to indicate that the *GLIS3* rs7034200C/A SNP is associated with the genetic predisposition of GDM, with Chinese women carrying the AA genotype and the A allele of the rs7034200C/A variant showing an elevated risk of developing GDM. Additionally, we found that the risk of GDM is higher in pregnant women with both *GLIS3* rs7034200AA and *ADRB3* rs4994TT genotypes than in those with the combined CC/TT genotype. We further found that the *GLIS3* rs7034200C/A polymorphism had a significant effect on fasting and 2-hour glucose concentrations in the OGTT during 24–28 weeks of pregnancy, HDL-C, fasting insulin, HOMA-IR, TOS, OSI, TAC, and TG/HDL-C ratio among GDM and/or control pregnant women. Our findings imply that the *GLIS3* rs7034200C/A genetic variant contributes to oxidative stress, insulin resistance, and glucolipid metabolism disorders.

Insulin resistance, islet β-cell dysfunction, and a relative shortage in insulin production are critical pathophysiological mechanisms in the occurrence and progression of GDM [2, 5]. GLIS3, a member of the GLIS subfamily of the Krüppel-like zinc-finger proteins, plays a significant role in the generation and maintenance of functional β-cells and insulin production; thus, it is a critical candidate gene for understanding the etiology and pathogenesis of GDM [11, 12]. *GLIS3* has 11 exons and expresses a protein of 930 amino acids [11]. Like other GLI and GLIS transcription factors, the *GLIS3* can recognize and bind specific DNA sequences of target genes via its DNA-binding domain, which contains five zinc-finger motifs, thereby regulating the transcription of these genes [11, 12]. Genome-wide association studies have shown that multiple SNPs in *GLIS3* are closely associated with several pathologies, including T2D and type 1 diabetes, in several populations [11, 12]. Among these SNPs, the *GLIS3* rs7034200 SNP, an intronic C/A variant, has been reported to be related to impaired fasting glucose, β-cell dysfunction, and T2D [11, 13], with the A allele or AA genotype exhibiting an elevated risk of T2D among the Chinese population [16, 17], South Asians [15], and a slightly or borderline increased risk in Europeans [18] and Japanese [32]. Conversely, this SNP was reported to be linked to a decreased risk of GDM in a dominant model in the Caucasian population [14]. In this study, we indicated that the A allele and AA genotype of the *GLIS3* rs7034200C/A SNP are associated with an elevated risk of GDM after adjusting for age, pre-pregnancy BMI, and gestational age at sampling among Chinese women. However, further adjusting for differences in glycolipid metabolic parameters, including HOMA-IR, LDL-C, and TG/HDL-C ratio, the dominant, recessive, and genotype models of this SNP no longer remained statistical significance, suggesting that this SNP may have a significant effect on glycolipid metabolism. Additionally, we found that patients carrying the *GLIS3* rs7034200 CA genotype exhibited lower TOS and OSI than those carrying the CC genotype, while those carrying the AA genotype had higher TOS than those carrying the CA genotype. Interestingly, we found that the controls with the AA genotype exhibited higher fasting and 2-hour OGTT glucose levels at 24–28 weeks of pregnancy, fasting insulin and TAC levels, and HOMA-IR than those with the CC or CA genotype. Furthermore, controls carrying the CA genotype exhibited a higher TG/HDL-C ratio but lower HDL-C levels than those carrying the CC genotype. Our findings indicate that the *GLIS3* rs7034200C/A variant significantly affects fasting and postprandial glucose, oxidative stress, fasting insulin, and lipid metabolism in the study population.

Increased pre-pregnancy BMI is closely linked to the onset and development of GDM. Human β3-adrenergic receptor (β3-AR) encoded by *ADRB3*, one of three β-adrenergic receptor members, is primarily expressed in adipose tissue. It binds to its specific ligand, catecholamine, and activates adenylate cyclase via its coupled G protein [20, 33]. The β3-AR-cAMP-dependent signaling pathway plays a significant role in regulating body fat distribution, lipolysis, thermogenesis, and metabolic rates [19–21]. The decreased expression of *ADRB3* is closely related to an increase in BMI and the occurrence of obesity [4, 22, 34]. Several factors, including activators of β3-AR (adrenaline, norepinephrine, and β3-AR agonists), inflammatory cytokines tumor necrosis factor α, high-fat diet, and obesity, have been proven to downregulate the expression of *ADRB3* through activation of exchange proteins directly activated by cAMP‌ (EPAC)/Ras-related protein Rab-2 A (RAP2A)/phosphoinositide-phospholipase C (PI-PLC) pathway, thus leading to catecholamine resistance in adipocytes [34]. The *ADRB3* rs4994 SNP, a T to C missense variant that leads to the substitution of tryptophan with arginine at codon 64, may cause structural changes in the ADRB3 protein and affect signal transduction [20, 33]. Several studies have reported a specific association between this genetic variant and ethnicity, gender, and disease status [19, 20, 35, 36]. The C allele (64Arg) carriers of rs4994T/C SNP significantly increase serum TG, TC, and leptin levels but decrease HDL-C and adiponectin concentrations mainly among obese Asian women [35], and are associated with an increased risk of essential hypertension in the Chinese and Caucasian population [37]. The rs4994T/C SNP has been widely studied for its association with T2D; however, findings have been inconsistent [20]. The association between the *ADRB3* rs4994 C allele and an increased risk of T2D, higher BMI, or adolescent and childhood overweight/obesity has primarily been observed in Asian populations [19, 20, 38]. The CC (Arg/Arg) genotype of this SNP was reported to be associated with higher annual BMI gain in Japanese men [39]. Our study found that the *ADRB3* rs4994T/C variant was not associated with GDM risk under different genetic models, and no significant discrepancies in clinical or biochemical indicators were observed between genotypes in the control and GDM groups. However, we observed that the combined AA/TT genotype of the *GLIS3* rs7034200C/A and *ADRB3* rs4994T/C SNPs was a risk factor for GDM when using the combined CC/TT genotype serving as a reference after adjusting for potential confounders. Further investigation is required to clarify the potential mechanisms underlying this association.

Our study has some limitations. We did not determine oxidative stress and metabolic indices in some participants due to hemolysis, high bilirubin levels, or insufficient specimen volume, which may have affected the statistical power for these parameters.

## Conclusions

This study demonstrated that the A allele and AA genotype of the *GLIS3* rs7034200C/A variant, along with the AA/TT combined genotype of the *GLIS3* rs7034200C/A and *ADRB3* rs4994T/C SNPs, were associated with an increased risk of developing GDM among pregnant Chinese women. We further demonstrated that the *GLIS3* rs7034200C/A variant contributes to insulin resistance, glucolipid metabolic disorders, and oxidative stress imbalance in the study population, thus may be involved in the pathophysiology of GDM. Our findings provide new evidence for elucidating the pathogenesis of GDM from a genetic perspective. Further research is needed to confirm the association between the *GLIS3* rs7034200C/A SNP and GDM in other ethnic groups and to elucidate the underlying pathophysiological mechanisms.

## Supplementary Information

Supplementary Material 1

Supplementary Material 2

Supplementary Material 3

## Data Availability

All data supporting the conclusions of this study are included within the article and its additional file. Further inquiries can contact the corresponding author.

## Abbreviations

ADRB3: Adrenergic receptor beta 3
Apo: Apolipoprotein
BMI: Body mass index
β3-AR: β3-adrenergic receptor
CI: Confidence interval
DBP: Diastolic blood pressure
GLIS3: GLI-similar 3
Glu: Glucose
Ins: Insulin
GDM: Gestational diabetes mellitus
HDL-C: High-density lipoprotein cholesterol
HOMA-IR: Homeostatic model assessment of insulin resistance
LDL-C: Low-density lipoprotein cholesterol
MDA: Malondialdehyde
OGTT: Oral glucose tolerance test
OR: Odds ratios
OSI: Oxidative stress index
SBP: Systolic blood pressure
SNP: Single-nucleoid polymorphism
T2D: Type 2 diabetes
TAC: Total antioxidant capacity
TC: Total cholesterol
TG: Triglyceride
TOS: Total oxidant status
