# Supplementary material for: GLIS3 rs7034200 and ADRB3 rs4994 genetic variants associated with an increased risk of gestational diabetes mellitus in Chinese women: a case-control study
Source: BMC Pregnancy Childbirth. 2025 Nov 21;25:1254. doi: 10.1186/s12884-025-08436-9 (PMC12639765; doi:10.1186/s12884-025-08436-9)
Supplement: Supplementary file 1 — Supplementary Material 1 [file 12884_2025_8436_MOESM1_ESM.pdf]

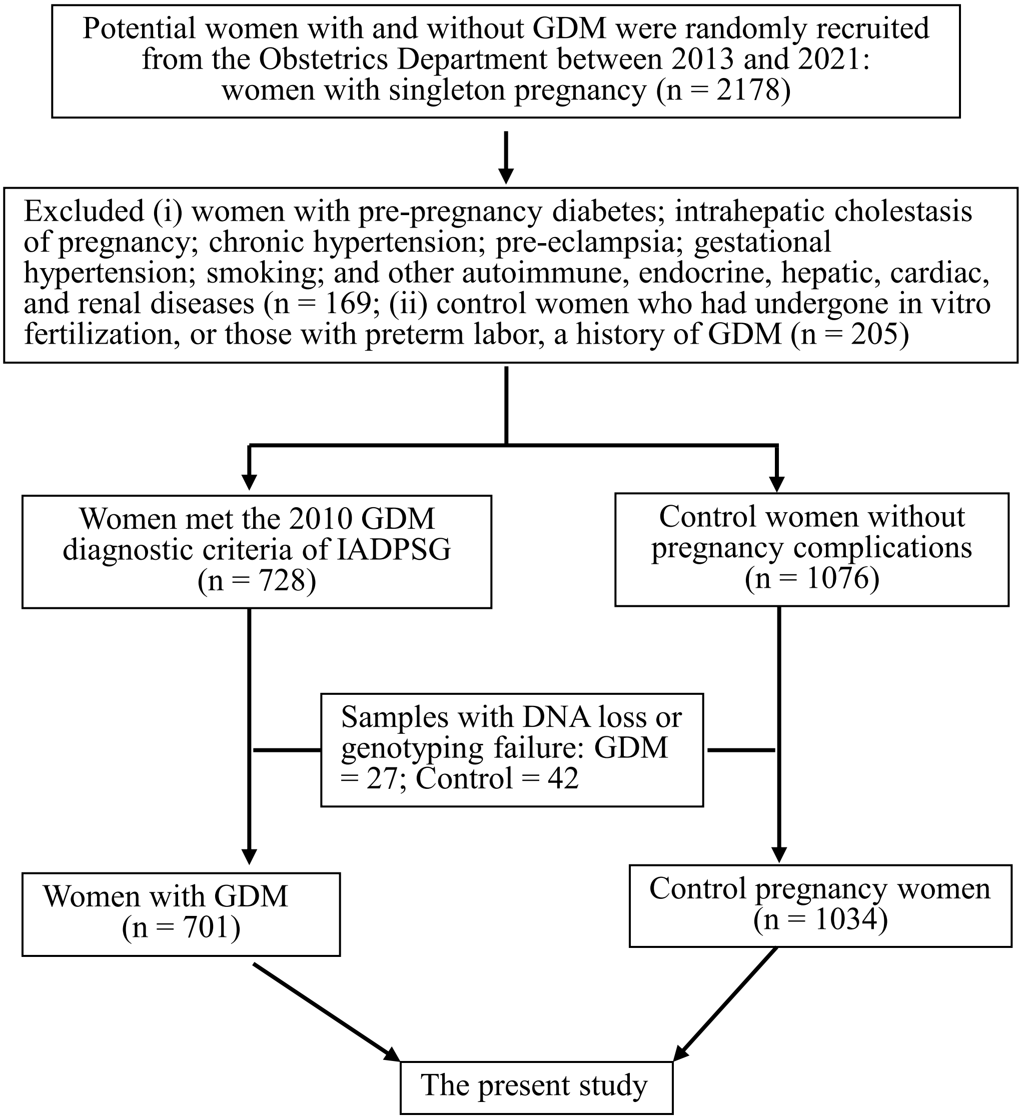

Supplemental Figure 1. Schematic diagram of the recruitment process of patients with GDM and control women
