# Supplementary material for: GLIS3 rs7034200 and ADRB3 rs4994 genetic variants associated with an increased risk of gestational diabetes mellitus in Chinese women: a case-control study
Source: BMC Pregnancy Childbirth. 2025 Nov 21;25:1254. doi: 10.1186/s12884-025-08436-9 (PMC12639765; doi:10.1186/s12884-025-08436-9)
Supplement: Supplementary file 2 — Supplementary Material 2 [file 12884_2025_8436_MOESM2_ESM.docx]

Supplemental Table 1. Effects of new adding covariates on the association between the *GLIS3* rs7034200C/A variation and the risk of GDM using multinomial logistic regression models

| Covariates | Recessive | |  | Dominant | |  | Genotype* | |
| --- | --- | --- | --- | --- | --- | --- | --- | --- |
|  | OR (95%CI) | *P* |  | OR (95%CI) | *P* |  | OR (95%CI) | *P* |
| Age, pre-pregnancy BMI, gestational age at sampling | 1.334  (1.020–1.745) | 0.035 |  | 1.268  (1.017–1.580) | 0.035 |  | 1.519  (1.120–2.061) | 0.007 |
| Age, pre-pregnancy BMI, gestational age at sampling,  pregnancy weight gain | 1.260  (0.955–1.664) | 0.102 |  | 1.267  (1.009–1.590) | 0.042 |  | 1.449  (1.057–1.986) | 0.021 |
| Age, pre-pregnancy BMI, gestational age at sampling,  HOMA-IR | 1.167  (0.866–1.571) | 0.310 |  | 1.140  (0.897–1.449) | 0284 |  | 1.320  (0.946–1.842) | 0.103 |
| Age, pre-pregnancy BMI, gestational age at sampling,  LDL-C | 1.248  (0.946–1.646) | 0.117 |  | 1.119  (0.956–1.504) | 0.117 |  | 1.372  (1.001–1.880) | 0.049 |
| Age, pre-pregnancy BMI, gestational age at sampling,  TG/HDL-C ratio | 1.251  (0.949–1.649) | 0.112 |  | 1.190  (0.949–1.492) | 0.133 |  | 1.369  (0.999–1.874) | 0.050 |
| Age, pre-pregnancy BMI, gestational age at sampling,  pregnancy weight gain, HOMA-IR, LDL-C  TG/HDL-C ratio | 1.122  (0.822–1.532) | 0.468 |  | 1.122  (0.873–1.443) | 0.369 |  | 1.268  (0.896–1.794) | 0.181 |

GDM, gestational diabetes mellitus; BMI, body mass index; HOMA-IR, homeostatic model assessment of insulin resistance; TG, triglyceride; HDL-C, high-density lipoprotein cholesterol; LDL-C, low-density lipoprotein cholesterol; OR, odds ratio; CI, confidence interval.

*** CC vs. AA genotype, CC genotype as the reference.
