# Supplementary material for: GLIS3 rs7034200 and ADRB3 rs4994 genetic variants associated with an increased risk of gestational diabetes mellitus in Chinese women: a case-control study
Source: BMC Pregnancy Childbirth. 2025 Nov 21;25:1254. doi: 10.1186/s12884-025-08436-9 (PMC12639765; doi:10.1186/s12884-025-08436-9)
Supplement: Supplementary file 3 — Supplementary Material 3 [file 12884_2025_8436_MOESM3_ESM.docx]

**Supplementary Table 2. Clinical and biochemical parameters according to *ADRB3* rs4994 different genotypes in women with and without GDM**

|  | Controls | |  | GDM | |
| --- | --- | --- | --- | --- | --- |
|  | TT  (n = 753) | TC + CC  (n = 260 + 21) |  | TT  (n = 505) | TC + CC  (n = 185 + 11) |
| **Clinical characteristics** |  |  |  |  |  |
| Age (years) | 35.44 ± 3.81 | 35.51 ± 3.61 |  | 35.41 ± 4.10 | 35.95 ± 4.03 |
| Pre-pregnancy BMI (kg/m^2^) | 21.25 ± 2.79 | 21.07 ± 2.40 |  | 22.32 ± 3.00 | 22.09 ± 2.84 |
| Delivery BMI (kg/m^2^) | 26.77 ± 2.78 | 26.48 ± 2.47 |  | 26.86 ± 2.98 | 26.74 ± 3.68 |
| Gestational age at sampling (wk) | 36.13 ± 3.54 | 36.11± 3.51 |  | 36.92 ± 3.28 | 37.30 ± 2.98 |
| Gestational age (wk) | 39.26 ± 0.86 | 39.28 ± 0.88 |  | 38.92 ± 1.15 | 39.05 ± 0.77 |
| Pregnancy weight gain (kg) | 14.11 ± 4.53 | 13.73 ± 3.59 |  | 11.49 ± 4.29 | 11.56 ± 3.84 |
| SBP (mmHg) | 114.93 ± 10.20 | 115.06 ± 9.89 |  | 115.95 ± 11.47  .01 | 115.46 ± 10.52 |
| DBP (mmHg) | 72.29 ± 7.75 | 71.85 ± 7.58 |  | 72.72 ± 9.28 | 72.70 ± 7.80 |
| OGTT-fasting Glu (mmol/L)* | 4.42 ± 0.30 | 4.44 ± 0.28 |  | 4.90 ± 0.53 | 4.87 ± 0.56 |
| OGTT-1 h Glu (mmol/L)* | 7.49 ± 1.30 | 7.34 ± 1.24 |  | 9.88 ± 1.39 | 9.93 ± 1.33 |
| OGTT-2 h Glu (mmol/L)* | 6.52 ± 1.02 | 6.56 ± 1.02 |  | 8.65 ± 1.35 | 8.77 ± 1.30 |
| Neonatal birth height (cm) | 49.86 ± 1.83 | 49.95 ± 1.99 |  | 49.56 ± 1.93 | 49.72 ± 1.62 |
| Neonatal birth weight (g) | 3375.40 ± 365.16 | 3415.90 ± 375.75 |  | 3320.37 ± 445.09 | 3370.38 ± 437.95 |
| **Metabolic parameters**** |  |  |  |  |  |
| Fasting Glu (mmol/L) | 4.37 ± 0.25 | 4.32 ± 0.44 |  | 4.58 ± 0.71 | 4.65 ± 0.80 |
| Fasting Ins (pmol/L) | 71.27 ± 33.96 | 74.01 ± 38.23 |  | 100.75 ± 122.87 | 113.23 ± 143.74 |
| HOMA-IR | 2.01 ± 1.04 | 2.09 ± 1.21 |  | 3.26 ± 5.42 | 3.79 ± 5.46 |
| TG (mmol/L) | 3.59 ± 1.35 | 3.71 ± 1.55 |  | 3.88 ± 1.58 | 3.96 ± 1.89 |
| TC (mmol/L) | 6.05 ± 1.08 | 6.10 ± 1.13 |  | 5.91 ± 1.08 | 6.06 ± 1.14 |
| HDL-C (mmol/L) | 2.00 ± 0.42 | 1.97 ± 0.41 |  | 1.96 ± 0.43 | 1.99 ± 0.44 |
| LDL-C (mmol/L) | 3.17 ± 1.01 | 3.20 ± 0.97 |  | 2.94 ± 1.00 | 3.04 ± 0.90 |
| TG/HDL-C | 1.89 ± 0.85 | 1.97 ± 0.95 |  | 2.10 ± 1.01 | 2.10 ± 1.29 |
| ApoA1 (g/L) | 2.38 ± 0.41 | 2.34 ± 0.41 |  | 2.30 ± 0.42 | 2.29 ± 0.42 |
| ApoB (g/L) | 1.14 ± 0.25 | 1.17 ± 0.28 |  | 1.14 ± 0.25 | 1.17 ± 0.27 |
| **Oxidative stress parameters***** | |  |  |  |  |
| TOS (μmol H2O2 Equiv./L) | 20.89 ± 7.03 | 22.07 ± 7.03 |  | 26.19 ± 10.84 | 25.11 ± 9.78 |
| TAC (mmol Trolox Equiv./L) | 1.10 ± 0.19 | 1.11 ± 0.21 |  | 1.13 ± 0.21 | 1.10 ± 0.20 |
| OSI | 19.28 ± 7.19 | 20.46 ± 7.54 |  | 23.42 ± 10.33 | 23.14 ± 9.58 |
| MDA (μmol/L) | 5.36 ± 1.21 | 5.43 ± 1.21 |  | 5.90 ± 1.42 | 5.82 ± 1.43 |

Values are presented as mean ± SD.

BMI, body mass index; DBP, diastolic blood pressure; SBP, systolic blood pressure; OGTT, oral glucose tolerance test; Glu, glucose; Ins, insulin; HOMA-IR, homeostatic model assessment of insulin resistance; TG, triglyceride; TC, total cholesterol; HDL-C, high-density lipoprotein cholesterol; LDL-C, low-density lipoprotein cholesterol; apoA1, apolipoprotein A1; apoB, apolipoprotein B; TOS, total oxidant status; TAC, total antioxidant capacity; OSI, oxidative stress index; MDA, malondialdehyde.

All parameter comparisons were corrected for differences in age, pre-pregnancy BMI, and gestational age at sampling between the two subgroups, except for age, BMIs, and gestational age at sampling.

*Plasma glucose levels during OGTT between 24 and 28 weeks of gestation.

**Controls (TT = 706, TC + CC = 246 + 20); GDM (TT = 462, TC + CC = 171 + 10).

***Controls (TT = 557, TC + CC = 189 + 14); GDM (TT = 380, TC + CC = 143 + 10).
